# Methylprednisolone as an Adjunct to Local Infiltration on Laminoplasty or Laminectomy before Wound Closure: A Randomized Controlled Trial

**DOI:** 10.1155/2022/2274934

**Published:** 2022-08-03

**Authors:** Niti Shrestha, Bo Han, Xiying Wang, Wenqing Jia, Fang Luo

**Affiliations:** ^1^Department of Pain Management, Beijing Tiantan Hospital, Capital Medical University, Beijing 100050, China; ^2^Department of Neurosurgery, Beijing Tiantan Hospital, Capital Medical University, Beijing 100050, China

## Abstract

*TrialDesign*. Patients undergoing laminoplasty and laminectomy often experience severe postoperative pain. Local infiltration analgesia during spine surgery significantly reduces postoperative pain, which only upholds for a short time. Whether methylprednisolone and local anaesthetics are better than local anaesthetics alone in postoperative analgesia is yet to be determined. The primary aim of this research was the postoperative evaluation of efficacy and safety of methylprednisolone when used as an adjunct to local anaesthesia, ropivacaine, before wound closure after surgical procedures, laminoplasty or laminectomy. *Methods*. 132 patients were divided with a ratio of 1 : 1 into methylprednisolone-ropivacaine and ropivacaine alone groups. Every 30 ml of local infiltration solution consisted of 15 ml of 1% ropivacaine with 14 ml of saline along with 1 ml of 40 mg methylprednisolone and 15 ml of 1% ropivacaine with 15 ml of saline in methylprednisolone-ropivacaine group and ropivacaine group, respectively. The standardization of the study solution depended on the number of levels involved in surgery. Primary outcome was the 48-hour cumulative sufentanil demand. *Results*. Demographic characters and surgical variables among the groups were identical. The average 48-hour cumulative sufentanil demand was 32.5 ± 20.6 *μ*g in the methylprednisolone-ropivacaine group and 50.9 ± 27.2 *μ*g in the ropivacaine group (*p* < 0.001). The estimated median time of demand of the first analgesia via patient-controlled analgesia (PCA) pump was 2.5 hours and 2 hours in the methylprednisolone-ropivacaine group and the ropivacaine group, respectively (hazard ratio (HR) was 0.53, with 95% Cl 0.33 to 0.87 and Log-rank of *p* = 0.0019). *Conclusion*. The infiltration of methylprednisolone as adjunct ropivacaine before wound closure is a safe and efficient strategy for pain management following laminoplasty or laminectomy.

## 1. Introduction

### 1.1. Background and Objectives

Patients undergoing laminoplasty and laminectomy often experience severe pain in the postoperative period [1, 2]. Suboptimal analgesic therapies cause discomfort to patients, prolong hospital stay, and increase health expenses [3], along with complications related to the reduction in patient mobility such as deep vein thrombosis [4, 5].

Postoperative pain following laminoplasty or laminectomy is a direct result of the dissection of muscles caused during these procedures [6]. Systemic medications are generally used to treat postoperative pain. In order to reduce the side effects of systemic medications, postoperative local analgesia has gradually become widely adopted for clinical use after spine surgeries.

The analgesia management and operational skill required for epidural and intrathecal anaesthesia following spine surgery are rather high [7]. Moreover, migration or kinking of epidural catheters that lead to catheter displacement and blockade, along with unpredictable analgesia, presence of blood in the epidural space, and drug absorption from the surgical site due to the breach in anatomical integrity after spine surgeries is of major concern [8]. Local infiltration analgesia (LIA) in spine surgery leads to a marked decrease in postoperative pain [9]. However, one major disadvantage of LIA is that the effect time is rather short.

Incisional infiltration on paravertebral muscles with bupivacaine plus methylprednisolone combined was reported to have provided similar pain control to that of bupivacaine alone after lumbar discectomy when administered preemptively [10]. However, the study only had 15 subjects in each group, with a total of 75 subjects and a short postoperative evaluation period of 24 hours. Another study established a considerable difference in postoperative analgesia demand and time of first analgesia demand via PCA in the local anaesthetic plus methylprednisolone group, when local infiltration of levobupivacaine-methylprednisolone and bupivacaine-methylprednisolone was used for one level lumbar disc surgery [11]. However, only 20 participants were included in each group with a 24 hours postoperative evaluation period. Hence, whether methylprednisolone and local anaesthetics are better than local anaesthetics alone in postoperative analgesia is worth exploring.

A recent systemic review [12] evaluated 39 RCTs (randomized controlled trials) and concluded that the preoperative or intraoperative and postoperative usage of paracetamol plus COX-2-specific inhibitor following lumbar laminectomy significantly advances postoperative pain management. The study also recommends intraoperative local wound infiltration or instillation of local anaesthetics to improve outcomes. Thus, we intended to establish a multimodal regimen with perioperative local wound infiltration of local anaesthetic combined with methylprednisolone before wound closure with the aim of achieving better results.

Therefore, we designed this prospective, randomized, open-label, blinded endpoint (PROBE) research to investigate our hypothesis that perioperative analgesia with the combination of methylprednisolone and ropivacaine before wound closure might result in better analgesic effects compared to ropivacaine alone after laminoplasty and laminectomy.

## 2. Methods

### 2.1. Trial Design

**Figure 1:**
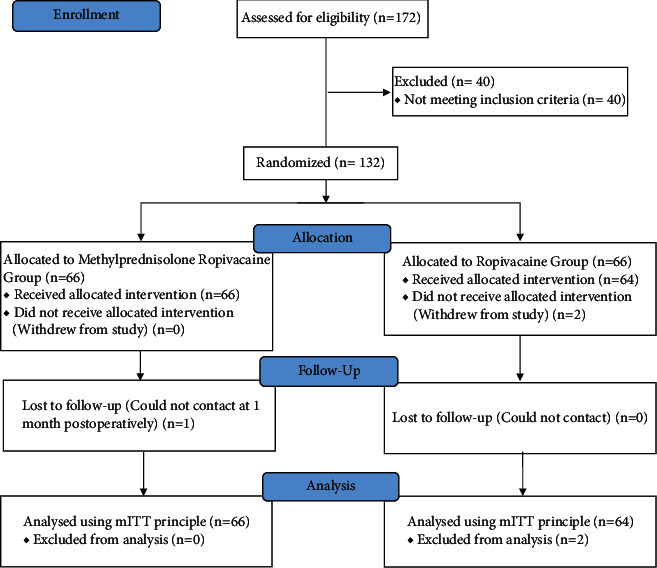
The CONSORT patient flow diagram showing numbers analysed in each group.

This was a PROBE, single-centre research, designed for the evaluation of the safety and efficacy of local methylprednisolone-ropivacaine infiltration against ropivacaine prior to closure of the incision for pain management following laminoplasty/laminectomy. 132 subjects were divided with a 1 : 1 ratio into methylprednisolone-ropivacaine (MR group) or ropivacaine (R group) group. The numbers analysed are shown in the CONSORT flow diagram as shown in Figure 1. The CONSORT 2010 checklist has been submitted as a supplemental file.

### 2.2. Participants

#### 2.2.1. Eligibility Criteria

- Elective laminoplasty/laminectomy under general anaesthesia.
- American Society of Anaesthesiologists (ASA) classification of I/II.
- Participants of ages between eighteen to sixty-four.

#### 2.2.2. Exclusion Criteria

- Denial from participation.
- Failure to provide written informed consent.
- Inability to understand or implement the concept of the Numeric Rating Scale (NRS).
- Failure to use patient-controlled analgesia (PCA) device.
- History of previous surgical procedure of the spine.
- Known history of previous allergic reaction to sufentanil, corticosteroids, or local anaesthetics.
- Unhealed infection at the incision site.
- Previous cerebrovascular accident (CVA).
- Old traumatic injury near the incision site.
- Psychological disorders.
- Body mass index (BMI) of less than 15 or more than 35.
- History of long-term opioid use of longer than 2 weeks, alcoholism, or drug abuse.
- Systemic steroids use.
- Gravid or lactating women.
- Glasgow Coma Scale of less than 15 preoperatively.
- History of or a possible need for radiation or chemotherapy before or after the procedure.

### 2.3. Study Setting, Ethics, and Recruitment

All research members received the same training protocol. The study design was registered on the clinicaltrials.gov website with registration number NCT04493463 and approved by the Institutional Review Board (IRB) of our hospital with approval number KY 2019-112-02-2 on 2019/11/16. The IRB conducted regular inspections of the trial progress. Subjects were enrolled from the neurosurgery outpatient department (OPD) of our hospital. An assistant visited each interested participant a day prior to the operation to explain the written consent in detail. Written informed consent was obtained on the day of surgery. Details regarding data sharing, withdrawal from the study, and so on were explained in the written informed consent.

## 3. Interventions

### 3.1. Anaesthesia Induction and Management

All participants were preoperatively familiarized with the concept of NRS to describe their degree of pain and were trained regarding the use of a PCA device after written informed consent was signed. Identical anaesthetic protocol and standard monitoring devices were used for both groups [5]. Induction of anaesthesia was done using midazolam 0.03 mg/kg, sufentanil 0.3 to 0.4 *μ*g/kg, propofol 1.5 to 2 mg/kg, and cisatracurium 0.2 mg/kg or 0.6 mg/kg rocuronium [13]. Mechanical ventilation was provided via endotracheal tube intubation with intravenous propofol 4 to 8 mg/kg/hr and remifentanil 0.1 to 0.3 *μ*g/kg/min [14]. To maintain mean arterial pressure and heart rate fluctuations within a 20% range of baseline, additional doses of vasoactive drugs were administered [2]. No additional analgesics were administered intraoperatively. The analgesic regimens in both groups were standardized. Local infiltration was administered by the neurosurgeon before wound closure. A complete record of drug dosage throughout the entire surgery along with all physiological parameters was maintained.

### 3.2. Local Infiltration

A 22 gauge needle was used to inject the incision site before wound closure. The number of levels to be treated determined the amount of study solution used. The study solution was not infiltrated into the epidural space and intrathecal space, whereas the spinous process, lamina, and transverse process were infiltrated along with the facet joints. Every 30 ml of study solution consisted of 1 ml of 40 mg methylprednisolone (Solu Medrol, injection methylprednisolone sodium succinate 40 mg, Pfizer Manufacturing, Belgium NV) and 1% ropivacaine (Nai Le Pin ® 10 mg·ml^−1^, AstraZeneca AB, Sweden) 15 ml with 14 ml of saline in the MR group [10, 11, 15] and 1% ropivacaine 15 ml with 15 ml normal saline in the R group.

### 3.3. Additional Interventions

Immediately postoperatively, 8 mg ondansetron was provided as a prophylaxis antiemetic [16]. Postoperatively, all patients were provided with a PCA pump (Apon® electronic infusion pumps ZZB-I-150, APON Medical Technology Co., Ltd., Jiangsu, China) with 1 *μ*g·ml^−1^ sufentanil for a period of 48 hours. All patients used the same model of PCA pumps, which was programmed to deliver only on demand, providing 2 ml with every press. Both initial dose and background infusion were set at zero. Every press delivered a 2 ml solution containing 2 *μ*g sufentanil, having a lockout period of 10 minutes. The maximum sufentanil dose was restricted to 8 *μ*g per hour. Participants pushed the analgesic demand button at a pain score of >4. If the maximum dose of sufentanil was received and the pain score was still >4, patients were to be consulted by neurosurgeons and pain physicians to decide whether to increase the maximum limit or to take another oral rescue analgesic.

If needed, participants were allowed to take oral analgesics, oxycodone and acetaminophen tablets (Mallinckrodt Inc.), a combination of 5 mg of oxycodone hydrochloride (equivalent to 4.4815 mg) and 325 mg paracetamol per tablet, every 6 hours, after 48 hours postoperatively, till the completion of our research.

### 3.4. Outcomes

Cumulative sufentanil dose recorded via PCA pump at postoperative 48 hrs was the primary outcome. NRS score during movement (NRS_M_) and rest (NRS_R_) were used to measure pain scores: an 11 points score in which “0” indicated nonexistent pain and 10 indicated the worst imaginable pain. NRS_M_ and NRS_R_ were recorded at hours 2, 4, 8, 24, and 48, day 3, and weeks 1, 2, and 4 postoperatively by in-person visits during the hospital stay and via telephone after discharge, by a blinded research member not involved in any other aspects of this research. Patient satisfaction scores (PSS) were also recorded along with NRS. PSS comprised 11 points, with 0 indicating not satisfactory and 10 indicating extremely satisfactory postoperatively. Postoperative nausea and vomiting (PONV) comprised nausea severity ranging from 0 to 3, in which 0 indicated none, 1 indicated mild not necessitating any treatment, 2 indicated necessitating treatment, and 3 indicated vomiting. PONV was recorded at hours 2, 4, 8, 24, and 48 postoperatively. Ramsay Sedation Scale (RSS) was used for measuring sedation levels and was recorded at the same time points as PONV postoperatively. RSS of 1 indicated agitated or anxious, 2 indicated cooperative, 3 indicated that the patient responded to commands only, 4 indicated that the patient responded strongly to glabellar tapping or loud external stimulation, 5 indicated that the patient responded weakly to glabellar tapping or noisy stimulants, and 6 indicated no response.

Wound healing score [17] was accessed at 4 weeks postoperatively, in which 3, 4-5, and 6+ indicated excellent, satisfactory, and suboptimal wound healing, respectively. Oswestry Disability Index (ODI) was assessed prior to surgery and 4 weeks after surgery. POSAS (Patient and Observer Scar Assessment Scale) was assessed 4 weeks after surgery.

### 3.5. Sample Size

Ersayli et al. [10] stated that cumulative morphine demand following lumbar discectomy in subjects receiving bupivacaine infiltration before wound closure was 13.4 ± 2.3 mg on the first postoperative day. The degree of analgesia provided by 1 mg morphine is equal to that by 1 *μ*g sufentanil. Hence, the morphine dose used by Erasyli et al. can be adapted for sufentanil (with conversion factor 1 mg morphine = 1 *μ*g sufentanil) as equianalgesic dose. Hence, total sufentanil consumption in our study was anticipated to be approximately 30.0 ± 9.0 *μ*g at 48 h after infiltration with local anaesthetics. Ersayli's study also reported how the addition of methylprednisolone reduces the dose of morphine demand by about 10–20% at the end of the first 24 h [10]. Thus, cumulative sufentanil demand during the first 48 h after lumbar discectomy was hypothesized to be 30.0 ± 9.0 *μ*g in the R group and 25.0 ± 7.5 *μ*g in the MR group. Therefore, on the basis of 90% power with a two-sided *α* of 0.05, we estimated that at least 59 participants were needed in each group. To account for a withdrawal of 10%, 132 participants had been enrolled in our study.

### 3.6. Randomization and Blinding

After written consent was obtained, subjects were allocated to either of the two groups in a 1 : 1 ratio with Statistical Package for the Social Sciences (SPSS 22.0). Instructions on the evaluation of pain and recovery were identical for both groups. The allocations were sealed in opaque envelopes and were only taken out on the day of surgery sequentially according to the order of participants. This was an open-label study design; only outcome assessors, data analysts, and postoperative pain evaluators were blinded.

### 3.7. Statistical Methods

Statistical analyses were done using SPSS 22.0. The calculation of the normality of variables was done using the Kolmogorov–Smirnov test. Mean ± SD (standard deviation) were used to present data for normal distribution. Median was used to describe variables for skewed distributions along with IQR (Interquartile Range). Frequencies with percentages were used to present categorical variables.

An independent *t*-test was used for comparison between normally distributed data. Mann–Whitney *U* test was used for comparison of skewed data. Categorical data were compared by the *χ*^2^ test or Fisher's exact test. The modified intention-to-treat (mITT) principle was used for primary analysis, and patients who did not receive the intervention were not included in the final analysis. Because of the exploratory nature of secondary outcomes, there was no multiple comparison adjustment of secondary analysis for *p* value. Comparison of time of demand of first analgesic demand via PCA was performed by univariable Cox proportional hazards model. Multiple imputation was used for handling missing data. The significance level was set at *p* < 0.05.

## 4. Results

### 4.1. Participant Flow and Recruitment

172 participants scheduled for elective laminoplasty or laminectomy under general anaesthesia were screened from July 31, 2020, to April 6, 2021. Forty participants were excluded. Both groups were randomly assigned 66 participants each, and 2 out of the 66 in the R group were excluded. During the final analysis, there were 66 and 64 patients in the MR group and R group, respectively. The CONSORT patient flow diagram is shown in Figure 1.

The CONSORT patient flow diagram shows numbers analysed in each group.

### 4.2. Baseline Data

There was no difference with regard to demographic data and surgical variables (Table 1). The average of total infiltration amount in both groups was 14.9 ml.

Summary of patient characteristics, including demographic data and surgical variables.

### 4.3. Outcomes and Estimation

#### 4.3.1. Primary Outcome

The 48-hour average cumulative sufentanil consumption in the MR group was 32.5 ± 20.6 *μ*g, and that of the R group was 50.9 ± 27.2 *μ*g (*p* < 0.001) as shown in Table 2.

#### 4.3.2. Secondary Outcome

**Figure 2:**
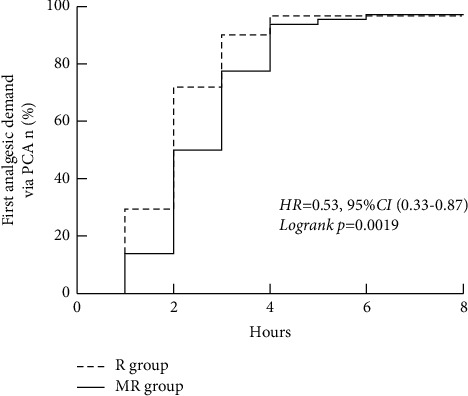
Comparison of estimated median time of analgesic demand via PCA between the MR (methylprednisolone plus ropivacaine) group and the R (ropivacaine) group.

Secondary outcomes have been demonstrated in detail in Table 2. The comparison of the estimated median time of analgesic demand via PCA between the MR (methylprednisolone plus ropivacaine) group and the R (ropivacaine) group is shown in Figure 2. The comparison of postoperative nausea and vomiting (PONV) along with Ramsay Sedation Score (RSS) between the MR (methylprednisolone plus ropivacaine) group and the R (ropivacaine) group within the 48 hours postoperative period is shown in Table 3. The comparison of wound healing scores, WHOQOL-BREF scores, POSAS scores, and ODI between the MR (methylprednisolone plus ropivacaine) group and the R (ropivacaine) group at 4 weeks is shown in Table 4. The raw data has been included as a supplemental file and will be available from the corresponding author upon reasonable request.

Comparison between primary outcomes and secondary outcomes among the MR (methylprednisolone plus ropivacaine) group and the R (ropivacaine) group in the first 48 hours postoperatively.

The estimated median time of first analgesic demand via PCA was 2.5 hours in the MR group and 2 hours in the R group. The hazard ratio (HR) was 0.53 with 95% Cl 0.33 to 0.87, and Log-rank was *p*=0.0019.

Comparison of wound healing scores, WHOQOL-BREF scores, POSAS scores, and ODI between the MR (methylprednisolone plus ropivacaine) group and the R (ropivacaine) group at 4 weeks.

### 4.4. Harms

A comparison of outcomes was performed for the safety assessment of postsurgical adverse events. Close monitoring of complications such as nerve injury, infection, and hematoma was done along with side effects including delirium, gastrointestinal (GI) bleeding, gastritis, and delayed wound healing due to the use of steroids.

## 5. Discussion

### 5.1. Limitations

We acknowledge several limitations of our research. Additional studies are required to assess the effectiveness of different concentrations of different types of steroids to determine the best possible outcome in short-term and long-term postoperative pain control. Future studies need to explore if preemptive surgical site infiltration could improve outcomes by inhibition of central sensitization before tissue trauma. Additionally, we were unable to find relevant references in which sufentanil was used for postoperative analgesia after spine surgery. Morphine versus sufentanil half-life and the natural overall decrease in pain intensity over time after surgery may have impacted the power value of this study. However, our research guarantees a power value of 99%, and therefore, the results of our study are credible.

According to the recommendations in PROSPECT guidelines for laminectomy [12], recommendations for preoperative and intraoperative analgesia include either oral or IV NSAIDs/COX-2-specific inhibitors (Grade A) and incisional wound instillation/infiltration by a local anaesthetic (Grade A). And recommendation for postoperative analgesia also includes either oral or IV NSAIDs/COX-2-specific inhibitors (Grade A). However, preoperative and intraoperative analgesic use are highly likely to influence pain scores and analgesic demand within 48 hours after surgery. Therefore, no analgesics were used prior to surgery, and no other analgesic drugs were used perioperatively except for routine maintenance anaesthesia. In order to ensure adequate analgesia for 48 hours postoperatively, along with the adjustment of the amount of sufentanil in the PCA device, neurosurgeons and pain physicians were also responsible for deciding whether to provide other oral rescue analgesics, if necessary. Although the analgesic principles of our research are identical to the PROSPECT guidelines for laminectomy, the specific analgesic regimen is rather different. This weakens the results of our study as they are not tested within an optimized regimen for pain control.

NRSM scores remained decreased in the MR group compared to the R group at 4 h, 8 h, 24 h, 48 h, 3 d, 1 w, and 2 w (*p* < 0.05), and there was a significant reduction in pain scores at movement and at rest. Nevertheless, the NRSM at 2 weeks in both groups has a median (IQR) of 0. The statistically significant result at this time point may have happened by chance and is not conclusive of the reduction of pain score. Although pain is an important factor determining patient satisfaction, other factors, including functional recovery and surgical outcomes, considerably influence satisfaction scores. Therefore, the results of this study must be acknowledged with caution. Moreover, even though the study confirms that an average of 19.9 ± 9.1 mg of methylprednisolone added to 14.9 ± 11.1 ml of 1% ropivacaine is safe, this result is unlikely to confirm these outcomes as the study is not powered to these secondary outcomes, and the adverse effects may have been rare to detect.

This was an open-label study design. Since methylprednisolone is not transparent when drawn in a clear syringe containing ropivacaine plus saline, it can be easily distinguished from a syringe containing only ropivacaine plus saline. Therefore, only the outcome assessors, data analysts, and research members responsible for postoperative pain evaluation were blinded. In future studies, an independent member of the research team will be accountable for prefilling the study solution in a nontransparent syringe so that surgeons and anaesthesiologists involved in the study will also be blinded. Moreover, to maintain consistency among the participants, strict exclusion criteria were used in this study, which may have limited the generalization of the study results. In the future, we plan to design more studies to explore the efficacy of this analgesic regimen in different clinical settings. Furthermore, there is also a possibility of undocumented self-medication with over-the-counter analgesics by patients in case of severe pain at home. Finally, this was a single-centre research; a larger scale multicentre trial will be beneficial in delivering more clinically substantial data.

### 5.2. Generalisability

This is the largest scale study of its kind with the longest follow-up period to date, which compares the postoperative analgesic efficacy of the local infiltration of methylprednisolone when added to local anaesthesia, ropivacaine, versus ropivacaine alone after laminoplasty or laminectomy before suturing of the incision. The main finding of the study is that the combination of methylprednisolone with local anaesthetic is better than local anaesthetic alone in postoperative analgesia.

In this study, the combined use of methylprednisolone and ropivacaine at wound closure reduced cumulative analgesic consumption via PCA within 48 hours postoperatively, significantly reduced pain scores at movement and rest, and significantly improved patient satisfaction scores. The results of our study were different from previous study [10], which showed no advantage in postoperative opioid requirement or pain control with the use of methylprednisolone and bupivacaine over bupivacaine alone for local infiltration. Consistent with another study, however [11], we found that first sufentanil consumption time was considerably later, and cumulative sufentanil requirement was also significantly reduced in the MR group.

This study only reports on the effects of local administration of methylprednisolone with ropivacaine at wound closure after surgery. In fact, postoperative pain after laminoplasty and laminectomy is not just confined to incisional pain. Mirzai and colleagues infiltrated the paraspinal muscles and subcutaneous tissues with 20 ml of 0.25% bupivacaine, soaked a portion of autologous fat for 10 minutes in 40 mg methylprednisolone, and positioned it over the exposed nerve root before wound closure after lumbar discectomy [18]. They found an immediate decrease in opioid consumption and pain postoperatively in comparison to the group that received 20 ml of 0.9% saline. Another study also reported that methylprednisolone alone, when placed on decompressed nerve root after soaking in collagen absorbable sponge, decreased postoperative back pain until the twelfth postoperative day [19]. In the future, to further optimize postoperative analgesia, the effects of local anaesthetic with methylprednisolone on the exposed nerve root are worth exploring.

### 5.3. Interpretation

Tissue trauma leads to the release of a large number of inflammatory factors such as prostaglandin, serotonin, and bradykinin, which are the main cause of acute postoperative incisional pain due to direct stimulation of peripheral nociceptors [20, 21]. Local application of methylprednisolone inhibits phospholipase A2 and decreases the release of inflammatory mediators and cytokines, which causes potent inhibition of inflammatory cascade and reduces incisional pain [22]. The half-life of methylprednisolone is 24 hrs; approximately 1/16th of its effect remains at the wound site 4 days postoperatively [23], which leads to a significant opioid sparing effect in the early postoperative period. Additionally, the analgesic effect of methylprednisolone remains for at least 2 weeks, perhaps due to early reduction in postoperative pain during the crucial first 9–12 hours of maximum postoperative pain [24], which is paramount for early mobilization, satisfaction, improved outcomes, and lower complications [25].

Local administration of steroids has been associated with corticosteroid-induced hypopigmentation, perilymphatic atrophy, skin and soft tissue atrophy, rupture of tendon, infection, sepsis, hypersensitivity, local calcifications, and steroid flare [26–30]. To avoid local side effects, steroids with shorter half-lives, suitable solubility, and potency must be used [26, 28, 31]; among those steroids, methylprednisolone is more widely used [32]. To be on the safe side, the lowest possible concentration of methylprednisolone injectable suspension was used based on previous studies [10, 11, 15]. Consistent with previous studies [33, 34], we found that the addition of methylprednisolone to local anaesthesia did not result in an increase in AEs; no association between steroid use and postoperative wound healing complications like infection or dehiscence were observed. Therefore, our study demonstrates that an average of 19.9 ± 6.8 mg of methylprednisolone added to 14.9 ± 11.1 ml of 1% ropivacaine is safe.

Although systemic steroids are gaining increased popularity in Enhanced Recovery After Surgery (ERAS), the benefits of local steroid infiltration have been well known since as early as 1999 when Dammers et al. used 40 mg methylprednisolone with 10 mg Lignocaine for Carpal Tunnel Syndrome and achieved tremendous results [35]. Systemic steroid use reduces the postoperative risk of PONV and acute pain [36]; however, a significant rise in blood glucose is also a known side effect of steroid use [37], along with sepsis, pneumonia, GI bleed, wound infection, and acute corticosteroid myopathy [38]. A multimodal analgesic regimen is widely used to improve outcomes, and the use of systemic and local corticosteroids as adjuncts has been suggested by previous studies [37]. Moreover, local steroid use had better results than oral systemic steroids in another report for the treatment of Carpal Tunnel Syndrome [39].

## 6. Conclusion

Perioperative analgesia with the combination of methylprednisolone and ropivacaine before wound closure results in additive or synergistic analgesic effects and reduces postoperative opioid consumption after laminoplasty and laminectomy, reduces postoperative pain, improves patient satisfaction scores without significant risks, and is expected to be one of the strategies for postoperative analgesia.

## Figures and Tables

**Table 1:** Summary of patient characteristics.

Characteristics	MR group (*n* = 66)	R group (*n* = 64)
Male/female	24/42	26/38
Mean age at the time of operation (yrs) ± SD	45. 0± 13.8	44.5 ± 13.0
Weight (kg) ± SD	66.6 ± 13.8	66.3 ± 11.2
Body mass index (kg/m^2^) ± SD	24.5 ± 4.4	24.2 ± 3.4
ASA status of (no. of patients) (I/II)	30/36	27/37
Preoperative pain intensity on a verbal Numeric Rating Scale (0–10), median (IQR)	6.0 (5.0, 7.0)	6.0 (5.0, 7.0)
Preoperative ODI	40.0 (40.0, 50.0)	42.0 (40.0, 54.0)
Level of spine treated (no. of patients (%)) Cervical Thoracic Lumbar Sacral Others (cervical + thoracic, thoracic + lumbar, lumbar + sacral, etc.)	22 (33.3%) 16 (24.2%) 12 (18.2) 2 (3.0%) 14 (21.2%)	30 (46.9%) 14 (21.9%) 12 (18.8%) 1 (1.6%) 7 (10.9%)
No. of levels treated (no. of patients (%)) 1 level 2 levels 3 levels 4 levels >4 levels	4 (6.1%) 20 (30.3%) 27 (40.9%) 11 (16.7%) 4 (6.1%)	10 (15.6%) 26 (40.6%) 16 (25.0%) 9 (14.1%) 3 (4.7%)
Amount of local anaesthetic (ml) ± SD	14.9 ± 5.1	14.9 ± 11.1
Dose of methylprednisolone (mg) ± SD	19.9 ± 6.8	0
Duration of surgery (h) ± SD	3.4 ± 1.3	3.0 ± 1.6
Duration of anaesthesia (h) ± SD	4.4 ± 1.3	3.9 ± 1.7
Duration of extubation (min) ± SD	13.0 ± 5.6	15.7 ± 9.0

**Table 2:** Comparison between primary outcomes and secondary outcomes among the MR (methylprednisolone plus ropivacaine) group and the R (ropivacaine) group in the first 48 hours postoperatively.

Time/variable	MR group (*n* = 66)	R group (*n* = 64)	*p* value
*Primary outcome*			
Cumulative sufentanil dose within 48 h (*μ*g) ± SD	32.5 ± 20.6	50.9 ± 27.2	<0.001

*Secondary outcomes*			
Cumulative sufentanil dose (*μ*g)			
0–4 h, ± SD	11.5 ± 7.1	15.6 ± 8.2	0.003
4–8 h, ± SD	9.8 ± 5.6	14.6 ± 7.4	<0.001
8–24 h, median (IQR)	6.0 (4.0, 10.0)	10.0 (8.0, 16.0)	<0.001
24–48 h, median (IQR)	4.0 (0, 6.5)	8.0 (4.0, 12.0)	<0.001

NRS_M_, median (IQR)			
2 h	6.0 (5.0, 8.0)	7.0 (6.0, 9.0)	0.116
4 h	6.0 (5.0, 7.0)	7.0 (6.0, 9.0)	0.006
8 h	5.0 (4.2, 7.0)	7.0 (5.0, 8.0)	<0.001
24 h	4.0 (3.0, 5.2)	5.0 (4.0, 7.0)	0.005
48 h	3.0 (2.0, 5.0)	5.0 (3.0, 6.0)	0.003
3 days	2.0 (1.8, 4.0)	3.0 (2.2, 5.0)	0.001
1 week	0 (0, 1)	1 (0, 2)	0.024
2 weeks	0 (0, 0)	0 (0, 0)	0.034
4 weeks	0 (0, 0)	0 (0, 0)	0.983

NRS_R_, median (IQR)			
2 h	3.0 (1.0, 4.0)	4.0 (2.2, 6.0)	0.007
4 h	2.5 (1.8, 4.0)	4.0 (2.0, 5.0)	0.001
8 h	2.0 (1.0, 3.0)	3.0 (2.2, 5.0)	<0.001
24 h	1.0 (0, 2.0)	2.0 (1.0, 3.0)	<0.001
48 h	0.5 (0, 1.0)	2.0 (1.0, 3.0)	<0.001
3 days	0 (0, 1)	1.0 (0, 2.0)	<0.001
1 week	0 (0, 0)	0 (0, 0)	0.061
2 weeks	0 (0, 0)	0 (0, 0)	0.076
4 weeks	0 (0, 0)	0 (0, 0)	0.983

PSS, median (IQR)			
2 h	4.0 (3.0, 6.0)	4.0 (2.0, 5.0)	0.078
4 h	5.0 (4.0, 6.0)	4.0 (2.2, 5.0)	0.007
8 h	5.0 (5.0, 7.0)	5.0 (3.0, 6.0)	0.001
24 h	7.0 (5.0, 8.0)	5.0 (4.0, 7.0)	0.003
48 h	8.0 (6.0, 8.0)	7.0 (6.0, 8.0)	0.002
3 days	8.0 (7.0, 9.0)	8.0 (6.0, 8.0)	0.001
1 week	10.0 (9.0, 10.0)	9.0 (9.0, 9.0)	<0.001
2 weeks	10.0 (10.0, 10.0)	10.0 (10.0, 10.0)	0.220
4 weeks	10.0 (10.0, 10.0)	10.0 (10.0, 10.0)	0.325

Number of participants who did not receive oral rescue analgesic after the 48-hour postoperative period	18 (27.3%)	20 (31.3%)	0.618

Oral rescue analgesics received after the 48-hour postoperative period (tablets)^*∗*^	0 (0–3)	0 (0–3)	0.715

^*∗*^Note: oxycodone and acetaminophen tablets (Mallinckrodt Inc.), each tab containing 5 mg oxycodone hydrochloride (equivalent to 4.4815 mg) and 325 mg acetaminophen.

**Table 3:** Comparison of postoperative nausea and vomiting (PONV) along with Ramsay Sedation Score (RSS) between the MR (methylprednisolone plus ropivacaine) group and the R (ropivacaine) group within the 48-hour postoperative period.

Time/variable	MR group (*n* = 66)	R group (*n* = 64)	*p* value
PONV, median (IQR)			
2 h	0 (0, 0)	0 (0, 0)	0.177
4 h	0 (0, 0)	0 (0, 1)	0.064
8 h	0 (0, 0)	0 (0, 0)	0.220
24 h	0 (0, 0)	0 (0, 0)	0.105
48 h	0 (0, 0)	0 (0, 0)	0.794

RSS, median (IQR)			
2 h	3.0 (3.0, 3.0)	3.0 (3.0, 3.0)	0.427
4 h	3.0 (2.0, 3.0)	3.0 (2.0, 3.0)	0.698
8 h	2.0 (2.0, 3.0)	2.0 (2.0, 3.0)	0.897
24 h	2.0 (2.0, 2.0)	2.0 (2.0, 2.0)	0.714
48 h	2.0 (2.0, 2.0)	2.0 (2.0, 2.0)	0.152

Comparison of postoperative nausea and vomiting (PONV) along with Ramsay Sedation Score (RSS) between the MR (methylprednisolone plus ropivacaine) group and the R (ropivacaine) group within the 48-hour postoperative period.

**Table 4:** Comparison of wound healing scores, WHOQOL-BREF scores, POSAS scores, and ODI between the MR (methylprednisolone plus ropivacaine) group and the R (ropivacaine) group at 4 weeks.

Time/variable	MR group (*n* = 66)	R group (*n* = 64)	*p* value
Wound healing scores at 4 weeks (no. of patients)			0.711
Suboptimal (6+)	6 (9.1%)	7 (10.9%)	
Very good (4-5)	57 (86.4%)	56 (87.5%)	
Excellent (3)	3 (4.5%)	1 (1.6%)	

WHOQOL-BREF scores at 4 weeks			
Psychological	13.1 (12.0, 14.2)	13.7 (12.6, 14.2)	0.160
Physical	13.7 (12.0, 14.7)	14.7 (13.3, 15.3)	0.057
Social	16.0 (14.6, 16.0)	16.0 (16.0, 16.0)	0.337
Environmental	16.0 (15.5, 16.1)	16.0 (16.0, 16.0)	0.190

POSAS scores at 4 weeks			
Objective scar rating	2.0 (2.0, 2.0)	2.0 (2.0, 2.0)	1.000
OSAS	8.0 (8.0, 8.0)	8.0 (8.0, 8.0)	0.106
Overall patient satisfaction	2.0 (2.0, 2.2)	2.0 (2.0, 3.0)	0.433
PSAS	9.0 (8.0, 9.0)	9.0 (8.0, 9.95)	0.913
ODI at 4 weeks	10.0 (8.0, 24.0)	12.0 (10.0, 19.5)	0.280

## Data Availability

The raw data will be available from the corresponding author upon reasonable request.

## Supplementary Materials

Supplementary Materials The raw data have been included as a supplemental file. The CONSORT 2010 checklist has also been submitted as a supplemental file.
